# Divergent Oxidative Stress in Normal Tissues and Inflammatory Cells in Hodgkin and Non-Hodgkin Lymphoma

**DOI:** 10.3390/cancers15133533

**Published:** 2023-07-07

**Authors:** Cecilia Marini, Vanessa Cossu, Francesco Lanfranchi, Sonia Carta, Francesca Vitale, Francesca D’Amico, Matteo Bauckneht, Silvia Morbelli, Maria Isabella Donegani, Silvia Chiola, Stefano Raffa, Luca Sofia, Tania Di Raimondo, Filippo Ballerini, Chiara Ghiggi, Paolo Durando, Silvia Ravera, Mattia Riondato, Anna Maria Orengo, Silvia Bruno, Sabrina Chiesa, Gianmario Sambuceti

**Affiliations:** 1 Institute of Molecular Bioimaging and Physiology (IBFM), National Research Council (CNR), 20054 Milan, Italy; 2 IRCCS Ospedale Policlinico San Martino, 16132 Genova, Italy; 3 Human Anatomy Section, Department of Experimental Medicine, University of Genoa, 16132 Genova, Italy; 4 Department of Health Sciences, University of Genoa, 16132 Genova, Italy; 5 Department of Internal Medicine, University of Genoa, 16132 Genoa, Italy

**Keywords:** lymphoma, redox stress, 2-NBDG, mitochondria, endoplasmic reticulum, pentose phosphate pathway, FDG-PET/CT

## Abstract

**Simple Summary:**

Background: Previous studies reported mitochondrial and endoplasmic reticulum (ER) redox stress in Circulating mononucleated cells (PBMCs) of patients with Hodgkin lymphoma (HL) display an oxidative damage to the endoplasmic reticulum. Here, we assessed whether the redox stress also characterizes tissues not directly involved in the inflammatory response and extends to non-HL (NHL) patients. Mitochondrial reactive oxygen species generation and malondialdehyde levels were increased only in the PBMCs of HL patients. These same cells also showed an enhanced activity of the hexose-6P-dehydrogenase (H6PD) and thus of a pentose phosphate pathway selectively confined within the ER. H6PD regulates the uptake of the most widely used tracer in clinical oncology: ^18^F-fluoro-deoxyglucose whose uptake was remarkably increased in the heart of HL patients. These data thus document that HL is associated with a high redox stress involving the ER. This feature does not apply to NHL. It is not limited to the PBMCs, and rather involves the myocardium as an epitome of tissues not participating to the inflammatory response to the disease.

**Abstract:**

Background: Previous studies reported mitochondrial and endoplasmic reticulum redox stress in peripheral blood mononucleated cells (PBMCs) of treatment-naïve Hodgkin lymphoma (HL) patients. Here, we assessed whether this response also applies to non-HL (NHL) patients, and whether the oxidative damage is a selective feature of PBMCs or, rather, also affects tissues not directly involved in the inflammatory response. Methods: Isolated PBMCs of 28 HL, 9 diffuse large B cell lymphoma, 8 less aggressive-NHL, and 45 controls underwent flow cytometry to evaluate redox stress and uptake of the glucose analogue 2-NBDG. This analysis was complemented with the assay of malondialdehyde (MDA) levels and enzymatic activity of glucose-6P-dehydrogenase and hexose-6P-dehydrogenase (H6PD). In all lymphoma patients, ^18^F-fluoro-deoxyglucose uptake was estimated in the myocardium and skeletal muscles. Results: Mitochondrial reactive oxygen species generation and MDA levels were increased only in HL patients as well as H6PD activity and 2-NBDG uptake. Similarly, myocardial FDG retention was higher in HL than in other groups as opposed to a similar tracer uptake in the skeletal muscle. Conclusions: Redox stress of PBMCs is more pronounced in HL with respect to both NHL groups. This phenomenon is coherent with an increased activity of H6PD that also extends to the myocardium.

## 1. Introduction

In patients with Hodgkin lymphoma (HL), peripheral blood mononucleated cells (PBMCs) are exposed to high redox stress even before chemotherapy most likely due to the immunomodulatory response [1,2]. However, whether this pattern also extends to tissues not directly involved in the inflammatory mechanisms has not been conclusively ascertained yet.

We have already shown that the increased oxidative stress in PBMCs of treatment-naïve HL patients largely involves the endoplasmic reticulum (ER) and is associated with an enhanced expression of hexose-6P-dehydrogenase (H6PD) [1]. This reticular enzyme is an autosomic homologue of the best-acknowledged glucose-6P-dehydrogenase (G6PD) and activates a pentose phosphate pathway (PPP) confined within the reticular lumen [3].

ER-PPP plays a pivotal role in the regeneration of NADPH reductive power in normal and inflammatory cells, as well as in cancer. Previous evidence documented that glucose flux through this reticular pathway largely regulates the cell uptake of the two de-oxygenated glucose analogues usually considered indicators of overall glucose consumption, namely: 2-[N-(7-nitrobenz-2-oxa-1,3-diazol-4-yl)amino]-2-deoxyglucose (2-NBDG) and ^18^F-fluoro-deoxyglucose (FDG) [4,5,6,7,8,9]. This paradigm thus links an imaging tool clinical used both to estimate tumor burden and therapy effectiveness coupling the evidence of an increased FDG uptake in normal tissues after chemotherapy for HL [10,11] with the reported increase in serum concentration of lipid peroxidation products such as malondialdehyde (MDA) [12].

Aiming to verify whether the accelerated redox stress of PBMCs is a generalized response [13,14,15,16], we thus verified whether the oxidative status of PBMCs predicts the myocardial uptake of FDG in patients with HL and non-Hodgkin lymphoma (NHL).

## 2. Materials and Methods

### 2.1. Patients

The study included 45 patients consecutively admitted to our institute from January 2020 to January 2023, for the diagnostic and staging evaluation of suspected, and subsequently confirmed, HL (n = 28), diffuse large B cell lymphoma (DLBCL, n = 9) or less aggressive NHL (follicular, n = 2 or mantle cell, n = 6 lymphoma less aggressive LA-NHL, n = 8). Exclusion criteria were a history of serious diseases, positivity for HBV, HCV and HIV, or any other coexistent condition asking for pharmacological therapy. As a control population, further 45 healthy subjects were recruited according to a case–control criterion among the participants to the periodical evaluations scheduled by the preventive medicine program of our IRCCS. All participants provided their written informed consent to participate in this study which was approved by the Ethical Committee of Regione Liguria (50/20-DB-id 10306).

### 2.2. PBMCs Isolation and Flow Cytometry Analysis

Blood samples (15 mL) were collected, and blood cell composition was characterized according to the same routine procedure of our Institute for both lymphocytes and monocytes. For control subjects and study patients, blood was obtained on the day of the control visit or PET imaging, respectively. The collected sample was then transferred into our laboratory to be analyzed within 24 h according to a previously validated procedure [17,18]. PBMCs were isolated using a density gradient separation medium (Lympholyte-H, Cedarlane, Burlington, ON, Canada), washed three times with Ca^2+^/Mg^2+^ free phosphate-buffered saline (PBS) and resuspended at 5 × 10^6^ cells/mL. Obtained cells were divided and dedicated to the different experimental evaluations.

In the first set, 5 × 10^5^ PBMCs were stained for 20 min at 37 °C with 5 μM MitoSOX Red, 10 μM the reactive oxygen species (ROS)-probe 2′,7′-dichlorodihydrofluorescein (DCF) or 50 μM 2NBDG (all from Invitrogen by Thermo Fisher Scientific, Waltham, MA, USA). The cells were then washed twice with PBS, centrifuged for 5 min at 290× *g* and resuspended in PBS + 1% BSA for flow cytometry analysis. Data were acquired on a FACSCan (Becton Dickinson, Milan, Italy) and data analysis was performed with FlowJo software (Version 9.96). The analysis of PBMC was restricted to viable cells, cellular viability was assessed by propidium iodide (PI) exclusion assays and cells positive for PI fluorescence were considered dead cells [19]. After gating procedures based on forward- and side-scatter features the recovery of lymphocytes and monocytes in the expected gate was confirmed by staining with CD3, CD19, and CD14 (Miltenyi Biotec, Bergisch Gladbach, Germany).

### 2.3. Seahorse Analysis

For this second set of experiments, 10^5^ PBMCs/well were seeded in XFp cell plates and centrifuged gently with no brake at 40× *g* for 3 min; the plate was then rotated 180° before centrifugation again at 80× *g* for 3 min to encourage adhesion to the plate and the forming of an evenly dispersed monolayer [20]. Oxygen consumption rate (OCR) and extracellular acidification rate (ECAR) were determined using the Seahorse XFp Extracellular Flux Analyzer (Agilent Technologies, Santa Clara, CA, USA). Cells were then incubated at 37 °C for 45 min in a CO_2_-free incubator with Agilent Seahorse DMEM, pH 7.4, enriched with glucose (11 mM). OCR and ECAR were monitored according to the manufacturer’s instructions. Briefly, three measurements of OCR and ECAR were taken under control conditions, after the injection of 1.5 μM oligomycin (ATP synthase inhibitor) and, thereafter, of 0.5 μM rotenone and 0.5 μM antimycin A to inhibit Complex I and III, respectively. According to the manufacturer’s instructions, ECAR was converted to proton efflux rate (PER) using WAVE software (Version 2.4, Agilent) after evaluating the buffer factor of the medium. To calculate the glucose flux through glycolysis, PER value (expressed in picomol H^+^ × min^−1^/million cells) of cells was converted to glucose nanomoles × min^−1^/million cells, applying the equation: glucose + 2 ADP + 2 Pi à 2 Lactate + 2 ATP + 2 H_2_O + 2 H^+^.

### 2.4. Enzymatic Assays

For this third set of experiments, PBMCs were again centrifuged at 300× *g* for five minutes and suspended in PBS supplemented by protease inhibitors to be sonicated twice for 10 s in ice, with a break of 30 s. The catalytic functions of glucose-6P-dehydrogenase (G6PD) and hexose-6P-dehydrogenase (H6PD) were assayed using an absorbance microplate reader (ELx808™, Winooski, VT, USA) to follow the reduction of NADP at 340 nm [4,6,7,8]. H6PD activity was tested in the presence of Tris-HCl pH 7.4 100 mM, glucose 10 mM, and NADP 0.5 mM. G6PD activity was assayed in the presence of Tris-HCl pH 7.4 100 mM, glucose-6-phosphate (G6P) 10 mM, and NADP 0.5 mM. In all cases, enzymatic activity was normalized for total protein concentrations tested using Bradford analysis [21]. MDA levels were evaluated, by the thio-barbituric acid reactive substances assay, using a UV/visible spectrophotometer (Ultraspec 2000, Pharmacia Biotech, Erie, PA, USA) [4,22].

### 2.5. FDG Imaging

PET/CT imaging was only performed in 45 lymphoma patients. According to current guidelines [23], a dose of 3–4 MBq of ^18^F-FDG was injected with the patient recumbent in a semi-supine position after a minimum of 6 h fasting and control of serum glucose level <150 mg/dL. Fifty minutes later, all scans were acquired according to the conventional procedure from the skull to mid-thigs, using a Hirez-16 PET/CT hybrid system (Siemens Medical Solutions). After diagnostic evaluation, an expert reader, unaware of the underlying disease, drew a volume of interest (VOI) over the left ventricular (LV) myocardium exploiting the CT scan in case of barely appreciable cardiac uptake. A further VOI was thus drawn over both psoas muscles, identified on the co-registered CT images at the midplane of the third lumbar vertebra body. In all cases, ^18^F-FDG concentration was conventionally estimated as both average and maximal standardized uptake value (SUV).

### 2.6. Statistics

All data were reported as mean ± standard deviation. One-way analysis of variance or unpaired t-test were used as appropriate to compare data in different groups. Linear regression analysis was performed by using the least squares method. A probability *p* < 0.05 was considered statistically significant. All statistical analyses were carried out by using dedicated software packages, namely, SPSS, v20 (SPSS, Chicago, IL, USA) and GraphPad Prism 9.3.1 (GraphPad, San Diego, CA, USA).

## 3. Results

### 3.1. Demographic and Hematologic Data

As shown in Table 1, control subjects, HL, DLBCL, and LA-NHL patients showed a similar prevalence of female gender and a comparable age. Likewise, routine evaluation of peripheral blood reported similar counts of red blood cells, platelets, white blood cells, granulocytes, lymphocytes, and monocytes. Within the HL cohort Ann Arbor scoring system is defined as class I (1), II (11), III (7), and IV (9) patients, respectively.

### 3.2. Redox Stress in PBMCs

**Figure 1:**
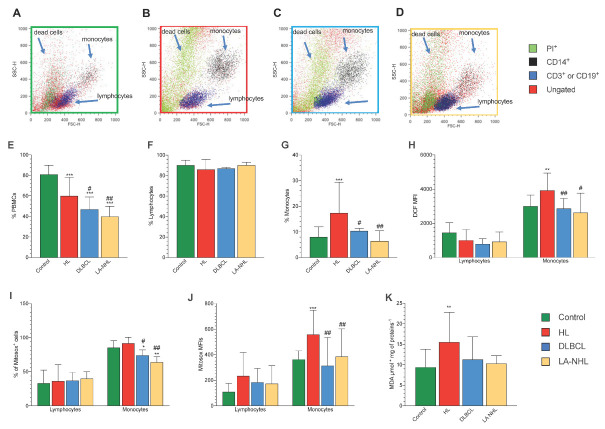
PBMCs cell culture, oxidative stress, and antioxidant response. Representative flow cytometry dot plots of forward- and side-scatter features (FSC, *x*-axis and SSC, *y*-axis) of PBMCs showing the recovery of lymphocytes and monocytes in the expected gates of controls (**A**), HL (**B**), DLBCL (**C**), and LA-NHL patients (**D**). Percentage of recovered viable PBMCs (**E**), lymphocytes (**F**), and monocytes (**G**) of controls (green), HL (red), DLBCL (blue), and LA-NHL patients (orange). Mean fluorescence intensity (MFI) of DCF (**H**) of lymphocytes (on the left) and monocytes (on the right). Percentage of MitoSOX-positive (**I**) and mean fluorescence intensity (MFI) of MitoSOX (**J**) of lymphocytes (on the left) and monocytes (on the right). Malondialdehyde content evaluated in lysed PBMCs (**K**). Data are expressed as mean ± SD. * = *p* < 0.05, ** = *p* < 0.01, *** = *p* < 0.001 vs. controls. # = *p* < 0.05, ## = *p* < 0.01 vs. HL group.

Flow cytometry (Figure 1A–D) analysis extended our previous observations in HL [1]. Indeed, the number of recovered viable PBMCs was highest in control samples, intermediate in HL, and lowest in both DLBCL and LA-NHL ones (Figure 1E). By contrast, although the relative prevalence of lymphocytes was not significantly affected by the presence of disease or its type (Figure 1F), recovered monocytes were represented with a higher prevalence in HL samples with respect to all other groups (Figure 1G). Intracellular ROS evaluation showed that DCF mean fluorescence intensity (MFI) was largely different according to cell type. Indeed, monocytes showed higher DCF-MFI values with respect to lymphocytes in all groups (*p* < 0.01, Figure 1H). However, HL monocytes showed a higher fluorescence with respect to all the other groups (Figure 1H). A slightly different pattern was displayed by mitochondrial redox status. Indeed, while the fraction of MitoSOX-positive lymphocytes was similar in all conditions (Figure 1I), NHL samples showed the lowest prevalence of positive monocytes with respect to both remaining groups (Figure 1I). On the other hand, HL monocytes displayed the highest fluorescent intensity of this mitochondrial probe while both DLBCL and LA NHL showed values similar to the control samples (Figure 1J).

Altogether, flow cytometry thus indicated that redox stress was decreased in NHL PBMCs. By contrast, mitochondrial ROS generation was selectively increased in HL monocytes. This difference was confirmed by the degree of oxidative damage estimated by the final product of polyunsaturated fatty acid peroxidation of PBMCs. Indeed, MDA levels were selectively increased in HL PBMCs with respect to both control and NHL samples (Figure 1K).

### 3.3. Energy Metabolism

Mitochondrial function represents, at the same time, a main ROS source and a primary target of redox-induced damage. We thus evaluated the energy metabolism of sampled PBMCs and the relative contribution of glycolytic flux and OCR, using the Seahorse technology. As for the evaluation of MDA content, this analysis was obviously limited to the unselected population of PBMCs, and no attempt was made to separate monocytes and lymphocytes.

**Figure 2:**
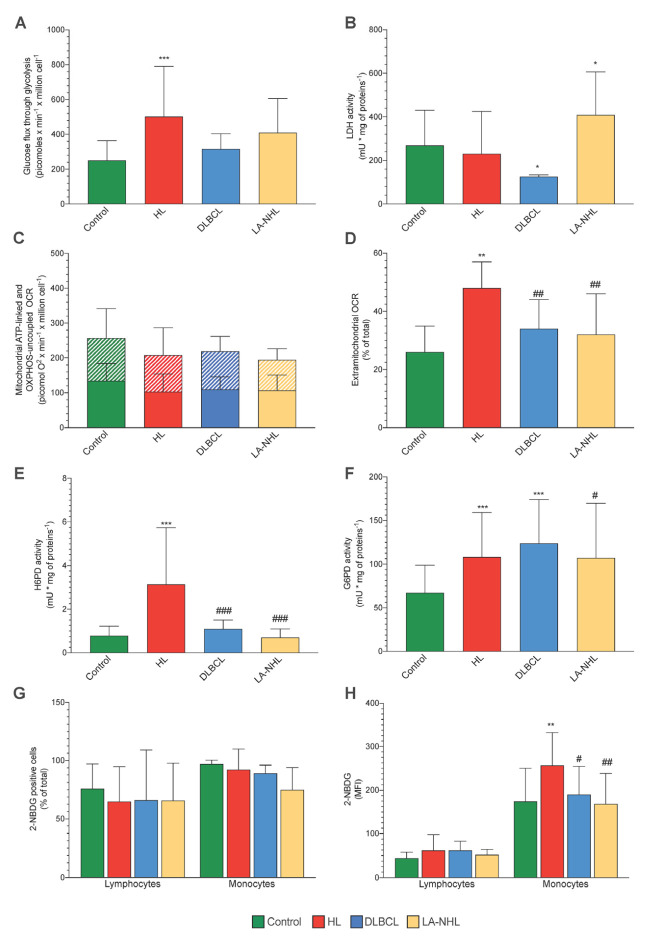
Mitochondrial energetic function and enzymatic activity in PBMCs. Glucose consumption through glycolysis in PBMCs (**A**) of controls (green), HL patients (red), DLBCL (blue), and LA-NHL patients (orange) at the Seahorse XFp analysis. Lactate dehydrogenase (LDH) activity of PBMCs (**B**). Mitochondrial ATP-linked OCR (solid-pattern) and OXPHOS–uncoupled OCR (dashed–pattern) (**C**). Extramitochondrial OCR, expressed as the percentage of the corresponding total OCR (**D**). Catalytic function of H6PD (**E**) and G6PD (**F**), measured by enzymatic assay in lysed PBMCs. Percentage of 2-NBDG-positive (**G**) and mean fluorescence intensity (MFI) of 2-NBDG (**H**) of lymphocytes (on the left) and monocytes (on the right). Data are expressed as mean ± SD. * = *p* < 0.05, ** = *p* < 0.01, *** = *p* < 0.001 vs. controls. # = *p* < 0.05, ## = *p* < 0.01, ### = *p* < 0.001 vs. HL group.

The functional evaluation showed a significant acceleration of glucose flux through glycolysis in HL PBMCs, although this increase did not reach statistical significance in DLBCL and LA-NHL (Figure 2A). This finding was not related to an increased rate of pyruvate conversion to lactate since LDH activity was not increased in HL samples while showing an opposite trend in the two NHL groups (Figure 2B). By contrast, both ATP-linked (oligomycin-sensitive) and OXPHOS-independent OCRs were comparable in all populations (Figure 2C). On the other hand, a greater fraction of OXPHOS-independent oxygen usage was warranted by extra-mitochondrial structures, particularly in HL PBMCs. Indeed, the ratio between rotenone–antimycin and total OCRs was significantly higher in HL PBMCs with respect to all the remaining samples (Figure 2D).

### 3.4. ER-PPP Activity and H6PD Abundance in Naïve HL and NHL Patients

The measurable oxidative damage displayed by the monocytes of HL samples was coherent with the previously reported enhancement in the catalytic function of both G6PD and H6PD in these patients with respect to healthy subjects [1]. The activation of both cytosolic and ER-PPP was indeed considered to indicate an accelerated NADPH regeneration to feed the reductive power needed to counterbalance the redox stress caused by this disease. In the present study, the activation of ER-PPP appeared to be a selective feature of HL. Indeed, H6PD activity was higher in these PBMCs with respect to all the remaining ones that were similarly low (Figure 2E). By contrast, a different behavior was documented for the cytosolic counterpart, since G6PD catalytic function was similarly increased in HL, DLBCL, and LA-NHL PBMCs with respect to control ones (Figure 2F).

Based on previous evidence [1], we thus aimed to verify whether the selective enhancement of H6PD activity was associated with an increased avidity of HL PBMCs for the fluorescent glucose analogue 2-NBDG. This hypothesis was indeed confirmed. The fractions of positive lymphocytes and monocytes were similar in all tested PBMC populations (Figure 2G). Nevertheless, the uptake of this fluorescent glucose analogue was significantly higher in the monocytes of HL patients with respect to all remaining PBMCs regardless of cell type or group of the sampled patient (Figure 2H).

### 3.5. PBMCs Redox Stress and Tissue FDG Uptake 

**Figure 3:**
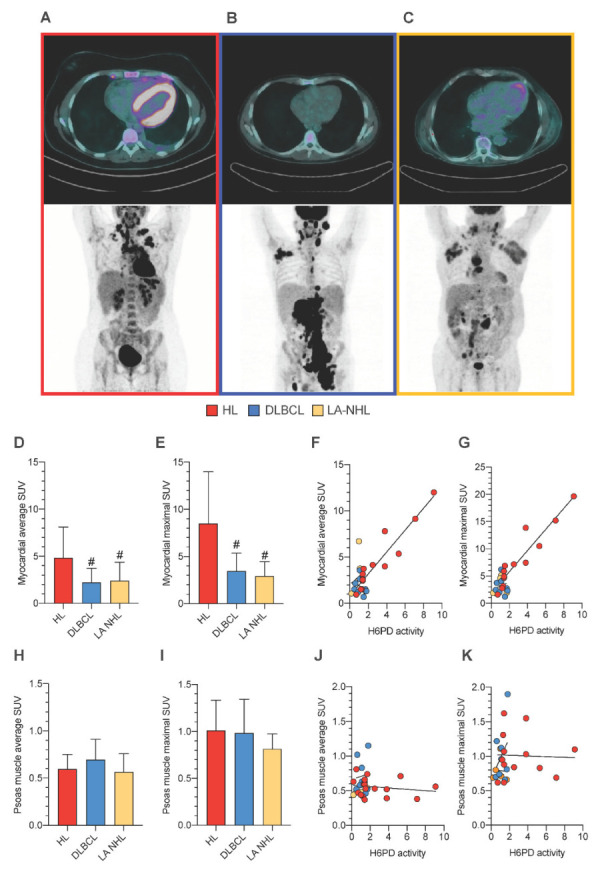
FDG uptake of the myocardium and the skeletal muscle in association with ER redox stress of PBMCs. Representative transaxial (above) and maximum intensity projection (below) images of two FDG-PET/CT scans performed in a patient with HL (**A**), in a patient with DLBCL (**B**), and in a patient with LA-NHL (**C**). Myocardial average (**D**) and maximal (**E**) standardized uptake value (SUV) in HL (red), DLBCL (blue), and LA-NHL patients (orange). Correlation between average (**F**) and maximal (**G**) SUV of the myocardium and H6PD activity in circulating PBMCs. Psoas muscle average (**H**) and maximal (**I**) SUV. Lack of correlation between average (**J**) and maximal (**K**) SUV of the myocardium and H6PD activity in circulating PBMCs. # = *p* < 0.05 vs. HL group.

Altogether, the analysis of PBMCs’ oxidative status indicated that the increased redox stress in HL PBMCs was associated with an enhancement in both extramitochondrial oxygen usage and glucose-6P flux through the ER-PPP. To explore whether this pattern was a selective response of PBMCs or rather a generalized phenomenon, we exploited the mandatory role of H6PD activity [11] to evaluate the FDG uptake in the myocardium and in the skeletal muscle. This analysis was obviously not performed in control subjects, while it was clinically indicated in all patients of the three lymphoma groups (Figure 3A–C).

Both average and maximal SUV were higher in the heart (Figure 3A–C) of HL patients with respect to both DLBCL and LA-NHL counterparts (Figure 3D,E). More importantly, FDG uptake and H6PD activity of PBMCs were evaluated on the same day in 18 patients in HL, 9 DLBCL, and 7 LA-NHL groups, respectively. When these subsets of patients were analyzed, both average and maximal SUV were tightly correlated with the activity of H6PD of PBMCs in HL patients. By contrast, this relationship was virtually absent in NHL subjects (Figure 3F,G).

Finally, the selectivity of cardiac response was documented by the evaluation of FDG uptake in the psoas muscles. Indeed, both the average and maximal SUV of this district were superimposable in all three groups (Figure 3H,I). Likewise, no relationship was observed between the metabolic pattern of this skeletal muscle and the H6PD activity of circulating PBMCs (Figure 3J,K).

## 4. Discussion

The data collected in the present study indicate more severe oxidative damage in the PBMCs harvested from HL with respect to DLBCL and other NHL patients. This damage was observed in treatment-naïve patients and thus cannot be attributed to the different chemotherapy protocols used in the two disease settings. The accelerated ROS generation of HL PBMCs was not limited to the mitochondria since their oxygen usage independent of Complex I and III activity was higher with respect to NHL counterparts.

By contrast, two observations indicate a measurable involvement of the ER in the generation or response to oxidative stress. On one side, HL PBMCs displayed an increased H6PD activity, suggesting an acceleration of glucose-6P flux through the ER-PPP, as opposed to the response of its G6PD-dependent cytosolic counterpart that was shared by both lymphoma types. On the other side, HL monocytes also displayed enhanced retention of the fluorescent glucose analogue 2-NBDG, whose accumulation kinetics has been previously documented to be strictly linked to the ER-PPP activation [4,5,6,7,8,9,10,11].

Altogether, these observations seemingly suggest that HL is associated with a higher inflammatory reaction with respect to both aggressive and relatively indolent NHL. This response extends to modifying the metabolic pattern of districts not directly involved in the inflammatory process. Indeed, the hearts of HL patients showed an increased FDG uptake that was evaluated by both average and maximal SUV to minimize the interference of tracer content in the arterial blood or its heterogeneous distribution [24]. The retention of this tracer is usually considered an index of tissue glucose consumption. Nevertheless, this classical model has been challenged by a series of studies documenting a strict link between FDG accumulation and H6PD catalytic function in cancer [6,8], in nervous tissue [7], in the skeletal muscle [5], and in the myocardium [11] that configured tracer accumulation as an index of ER-PPP activity and, thus, of the regeneration rate of NADPH reductive power within the reticular lumen.

To verify this hypothesis, we extended the analysis of tracer accumulation to the skeletal muscle, epitomized by the psoas. This district was chosen to minimize the possible interference by contractile activity on tissue metabolism during the time elapsing between tracer injection and image acquisition and, thus, to document the absent interference of nutritional status and insulinemia on myocardial tracer uptake.

Matching its high susceptibility to oxidative damage, the myocardium entails powerful antioxidant mechanisms. In agreement with previous studies [11], the enhanced cardiac FDG uptake thus suggests that cardiac response to HL-related redox stress largely involves the regeneration of NADPH reductive power by accelerating the glucose flux through the ER-PPP. Indeed, both cardiac SUV indexes (maximal and average) were directly correlated with the enhancement of the H6PD catalytic function of the harvested PBMCs. In addition to the acknowledged reticular confinement of H6PD [3], the involvement of reticular redox status in PBMCs of HL patients was further supported by the increased uptake of the FDG fluorescent analogue 2-NBDG. Indeed, the increased 2-NBDG MFI, observed in HL monocytes, was independent of glycolytic flux directly estimated by the Seahorse approach, and indirectly testified by the behavior of LDH activity. Altogether, these data thus suggest that HL is associated with signals, derived by either neoplastic or inflammatory cells, able to induce a generalized shift in the metabolic pattern and also in tissues not directly involved in the inflammatory process.

The enhanced redox stress of PBMCs at the staging phase of HL might have important implications for chemotherapy planning. This finding can hardly be attributed to the presence of a high number of circulating neoplastic cells and, thus, indicates that the oxidative damage extends to tissues and cells not affected by the disease even before the occurrence of treatment-related toxicity. This observation might possibly predict the risk for the oxidative damage induced in HL patients by several chemotherapeutic drugs, particularly to the myocardium, in agreement with the previous evidence about a significant predictive power of increased myocardial uptake of FDG after the first two cycles of doxorubicin therapy [25].

### Limitations

Several limitations of the present study should be carefully considered. As a first point, the heterogeneous distribution of NHL patients prevented an accurate evaluation of indolent NHL forms. Accordingly, the present data do not cannot be considered that NHL is per se a disease characterized by a scarce oxidative stress to normal tissues. Similarly, the limited sample size of the three groups obviously implies the need for further investigations to verify whether the divergent endurance of monocytes and lymphocytes against redox stress contributes to predicting the disease aggressiveness and its response to chemotherapy.

As a further limitation, several variables (including enzymatic activity, MDA content, OCR, and glycolytic flux) could not be separately investigated for the lymphocytes and monocytes because of the limited amount of available blood cells in these patients. These same limitations prevented applying the whole experimental set of evaluations in all patients.

Finally, the present study does not elucidate the molecular mechanisms underlying the enhanced redox stress in the PBMCs of untreated HL patients. This task would have implied a preliminary evaluation of cultured PBMCs of both groups, using media with content reproducing nutrients and signals present only in the peripheral blood environment of patients. This is a complex task considering the variegate effect affecting the host healthy cells and their unknown link with the lymphoma type.

## 5. Conclusions

The present study confirmed the previously observed redox stress in the non-neoplastic PBMCs of HL patients [1]. This response was not observed in both DLBCL and LA-NHL samples and cannot be attributed to the contamination of circulating blood by neoplastic cells. Thus, it indicates that HL is most likely associated with a particularly pronounced inflammatory reaction. The extension of this response to the myocardium implies the presence of signals released either by HL itself or by the inflammatory reaction it activated. This generalized shift in systemic metabolic pattern has been already found to retain a potential role in predicting anthracycline effectiveness [25]. Understanding the underlying mechanisms might open new windows to monitor cancer response to chemotherapy.

## Figures and Tables

**Table 1:** Demographic and routine data.

	Control Subjects	HL	DLBCL	LA-NHL
Gender (M/F)	24/21	(17/11)	(6/3)	(4/3)
Age	53 ± 12	54 ± 19	69 ± 16	65 ± 11
Isolated PBMC (×10^6^)	20.2 ± 10.3	26.1 ± 19.5	20.2 ± 1.1	21.1 ± 11.7
RBC (×10^6^)	4.6 ± 2.2	4.8 ± 0.6	4.5 ± 0.3	4.2 ± 0.7
HB (g/L)	141.2 ± 16.2	129.2 ± 15.7	126.2 ± 15.5	117 ± 22
HCT (%)	38.45 ± 6.2	39.2 ± 4.6	37.9 ± 4.8	34.8 ± 5.8
PLT (×10^3^)	287.5 ± 116.2	295.2 ± 121.1	270.1 ± 144.9	214.5 ± 138
	±			
WBC (×10^3^)	9.8 ± 4.2	9.4 ± 3.5	7.6 ± 2.8	9.6 ± 5.7
Granulocytes (×10^3^)	4.2 ± 2.1	6.9 ± 3.4	5.6 ± 2.1	4.5 ± 2.7
Lymphocytes (×10^3^)	1.5 ± 0.4	1.6 ± 0.7	1.2 ± 0.8	3.1 ± 2.4
Monocytes (×10^3^)	0.6 ± 0.4	0.7 ± 0.3	0.7 ± 0.3	0.5 ± 0.2
Mono/Lympho	0.5 ± 0.4	0.5 ± 0.4	0.93 ± 1.3	0.3 ± 0.2

HL: Hodgkin lymphoma; DLBCL: diffuse large B cell lymphoma; LA-NHL: less aggressive non-Hodgkin lymphoma; M: males; F: females; PBMC: peripheral blood mononuclear cell count; RBC: red blood cell count; HB: hemoglobin: HCT: hematocrit; PLT: platelet count; WBC: white blood cell count.

## Data Availability

Data is contained within the article.
